# Statistical analysis of residual chlorine distribution in Binh Tan District’s water network: insights for urban water management

**DOI:** 10.1098/rsos.251068

**Published:** 2025-08-20

**Authors:** Begmyrat Kulmedov, Emre Eroglu, Nguyen Huy Cuong, Tran Van Loi, Nguyen Hoan Phuong Thy

**Affiliations:** ^1^ Department of Civil Engineering, Epoka University, Tirana, Albania; ^2^ Ho Chi Minh City University of Natural and Resources Environment, Ho Chi Minh City, Ho Chi Minh City, Vietnam

**Keywords:** water distribution, residual chlorine, principal component analysis, regression modelling, hierarchical clustering, urban water management

## Abstract

This study examined the water distribution system in Binh Tan District, Ho Chi Minh City, Vietnam, focusing on residual chlorine levels across seven sampling points. Chlorine decay patterns and relationships between sampling points were investigated using statistical analyses, including reliability testing, correlation analysis, factor analysis and regression modelling. The study identified three principal components that explained 92.558% of the total variance, with strong correlations between specific sampling points. Five regression models were developed, which demonstrated high predictive power (*R*² ranging from 0.750 to 0.962) and reliability (Cronbach’s alpha 0.749–0.967). Hierarchical cluster analysis revealed distinct groupings of sampling points, providing insight into the spatial patterns of chlorine distribution. These findings not only provide insights into water quality management but also offer a transferable modelling framework for optimizing monitoring and chlorination strategies in other urban distribution systems.

## 1. Introduction

Maintaining adequate residual chlorine (RC) levels in water distribution networks is crucial to ensure safe drinking water. The World Health Organization (WHO) recommends RC concentrations between 0.2 and 0.5 mg l^−1^ for potable water, with higher levels (1.0−2.0 mg l^−1^) during emergencies [1,2]. Understanding the levels of free RC at various points within a water distribution network is essential for assessing the quality of the water delivered to consumers. RC levels are not uniformly distributed across the network because they are influenced by factors, such as chlorine decay rates, the supply system in place and the distance of distribution nodes from the source. The deterioration of RC levels is particularly pronounced at dead-end nodes, where low flow velocity and long residence times contribute to significant disinfectant decay [3]. A continuous water supply system is more advantageous than an intermittent one because it better preserves water quality and maintains consistent pressure throughout the network [4].

Network structure plays a crucial role in influencing water age and RC levels. In this regard, studies showed that strongly interconnected network structures (i.e. with a high number of loops) are optimal in terms of supply service and system reliability but can result in a reduction of the average velocity in pipes and possible problems related to water quality. In response to that, the tendency to close valves and create branched structures emerged and several approaches have been proposed to identify the optimal interventions on the topological structure of the network. For example, Abraham *et al.* [5] proposed to reduce the level of interconnection by closing a determined set of isolation valves to ensure optimal velocity conditions. At the same time, it emerged that drawbacks are related to the creation of branched structures in the networks. For example, topology modifications are confirmed to have an impact on the transient response of water distribution networks. As shown in Marsili *et al.* [6], as the number of branches increases and the number of interconnections and loops decreases, the pressure stress state of the network is emphasized.

Modelling chlorine decay in water distribution networks (WDNs) is essential for maintaining disinfection efficacy and ensuring safe water quality. Various approaches integrate both hydraulic and quality modelling to simulate chlorine residuals accurately. Moghaddam *et al.* [7] utilized particle swarm optimization to calibrate models based on observed pressure and chlorine levels, effectively minimizing simulation errors. Monteiro *et al.* [8] demonstrated that both first-order and advanced EXPBIO models could simulate wall decay reliably, with the simpler model often sufficient under high chlorine concentrations. Zaghini *et al.* [9] proposed an interval-based method that infers decay parameters in complex, multi-source networks using trace analysis, offering a practical solution where detailed data are lacking. Obaid *et al.* [10] applied Arrhenius-based decay modelling calibrated with field tests and smart meter data, enabling hourly estimation of low-chlorine risk zones using EPANET.

The deterioration of RC levels has been shown to correlate strongly with distribution distance, pH and temperature. For instance, in a study conducted in Malang, Indonesia, 65% of the sampling points in an isolated residential network showed RC values below 0.2 mg l^−1^, with regression analysis revealing significant negative correlations between chlorine levels and both pH and temperature [11]. Similarly, in Bishoftu Town, Ethiopia, a temporal and spatial chlorine decay analysis using WaterCAD simulation and laboratory data revealed a decrease in RC levels over time, highlighting water age as a critical factor influencing chlorine stability [12]. Similar modelling approaches have also been used to assess chlorine decay behaviour in complex distribution systems using EPANET-based simulations, highlighting the role of system layout and booster station placement in maintaining acceptable chlorine concentrations [13].

Operational strategies also play a key role in chlorine management. Phan *et al.* [14] addressed RC maintenance challenges by installing an additional chlorine station and successfully increasing and stabilizing the chlorine concentrations. This compact solution can be applied to other systems facing similar issues. However, another innovative solution involves modulating nodal outflows at critical points in the network, which was shown to improve RC levels without increasing source dosage or requiring new booster stations, thereby improving cost-efficiency [3].

Recent studies in Vietnam have highlighted the variations in RC levels across water supply systems. Kim & Tuyet [15] found that 92.9% of samples in Quang Ngai Province met standards, but note that effectiveness varied along distribution pipelines. Le *et al.* [16] observed significant variations across provinces, with some areas falling below the 0.2 mg l^−1^ limit. These inconsistencies may contribute to microbial contamination, as indicated by the weak correlations between chlorine levels and microbiological parameters [17]. Dat *et al.* [18] investigated disinfection by-products (DBPs) in Ho Chi Minh City’s water supply and found higher levels in certain zones and seasonal variations. This study revealed correlations between DBPs and water quality parameters, including RC, highlighting potential health risks [18]. These findings underscore the importance of maintaining consistent chlorine levels throughout the water distribution networks.

The objective of this study is to analyse chlorine dynamics and develop a predictive model to enhance monitoring efficiency in Binh Tan’s urban water distribution network. This research breaks new ground by using a comprehensive statistical approach, integrating spatial and temporal dimensions through reliability testing, correlation analysis, factor analysis, hierarchical clustering and regression modelling. The resulting predictive models and insights into chlorine behaviour represent significant advancements in water quality management. By bridging theoretical analysis with practical application, this study provides a novel framework for optimizing monitoring strategies and chlorination practices in rapidly growing urban areas, contributing valuable tools for water managers and policymakers. Although the analysis is focused on Binh Tan, the methodological approach is adaptable to other urban WDNs, allowing for broader application beyond the study area.

## 2. Material and methods

### 2.1. Study area

Binh Tan, an urban district of Ho Chi Minh City, Vietnam, was selected as the study area. Formed by the amalgamation of three communes and a town, Binh Tan is notable for being the most populous district in Ho Chi Minh City, with a population of 784  173 as of 2019. Covering an area of 50.02 km², it boasts a high population density of 15 074 people km^−^². The geographical and demographic characteristics of the district make it an ideal case study for urban water management purposes. Data on the district’s area, population and water supply coverage were obtained from the Statistical Yearbook of Vietnam [19] and recent studies [20,21], providing a comprehensive overview of the current situation.

### 2.2. Water treatment and distribution system

The water treatment and distribution system in the Binh Tan District was examined in detail. The primary water source for the district is the Saigon River [22], with water extracted and treated at a local facility capable of processing 300 000 m³ day^−1^ [23]. The treatment process involves several stages, including screening, chemical treatment with lime and chlorine, sedimentation using poly aluminium chloride (PAC), sand filtration and final chlorine disinfection [24]. The distribution system comprises a clean water pumping station with a capacity of 420 000 m³ day^−1^, factoring in the peak demand periods. The main water supply pipeline, a 1500 mm diameter prestressed concrete pipe stretching 11.3 km, connects the Tan Hiep Water Treatment Plant to the city’s network and Binh Tan District [21]. Information on the capacity of the system, treatment processes and infrastructure was collected from technical reports [24] and operational data provided by the Cho Lon Water Supply Joint Stock Company [25], which manages the district’s water supply network.

### 2.3. Data collection

**Figure 1.**
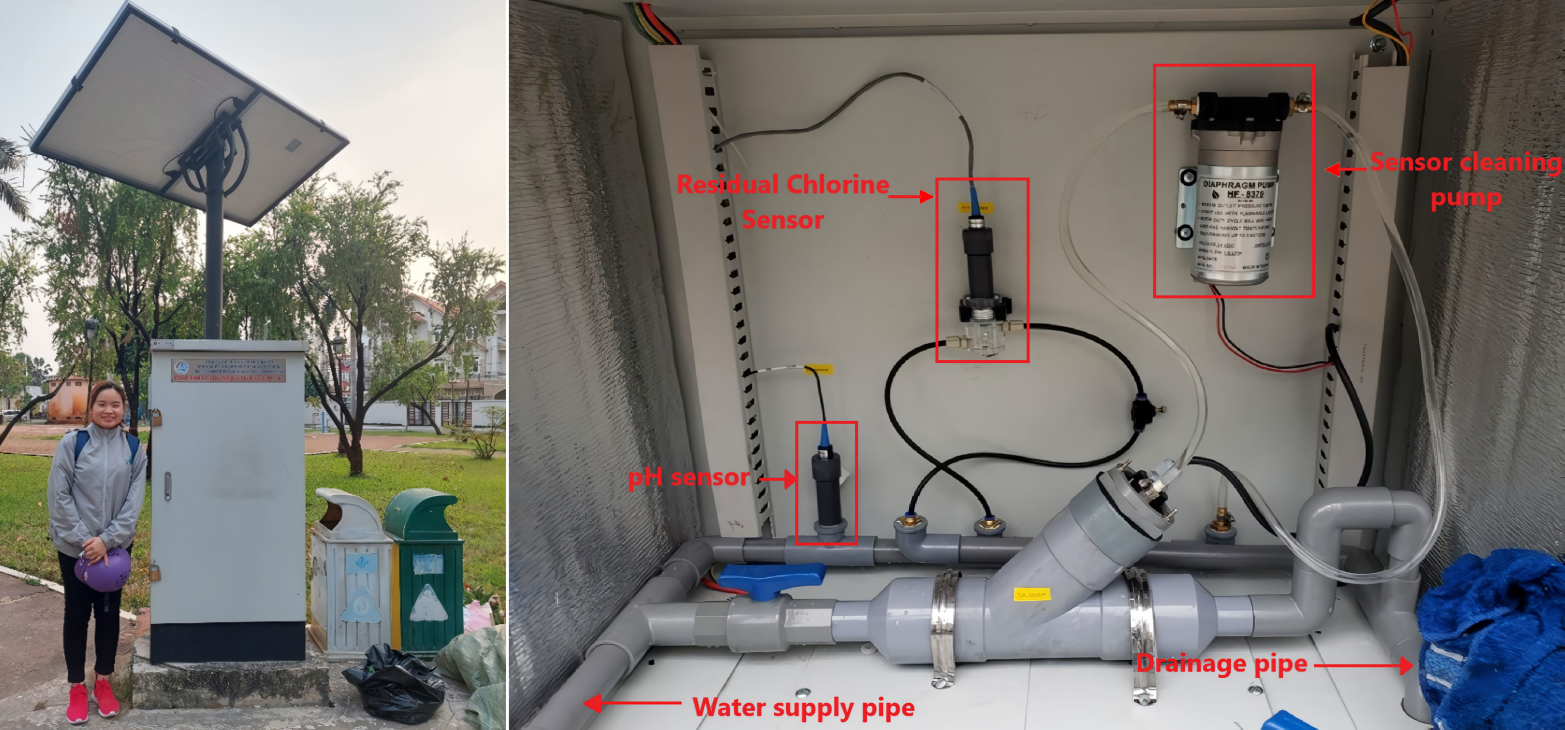
Water quality monitoring station.

In this study, two certified methods were used to measure RC. Sampling points water sampling point 1 ((WS1) Tan Hiep Water Treatment Plant), WS2, WS5 and WS6 were tested using the Hach DR300 portable chlorine test kit (USA), administered by the Quality Assurance and Testing Center 3 (QUATEST 3). Sampling points WS3, WS4 and WS7 were monitored through in-line automatic sensors installed at key junctions as shown in figure 1. Both instruments are approved for official water quality monitoring by Vietnamese regulatory authorities. To ensure data consistency, a pre-sampling calibration exercise was performed in which both devices measured chlorine levels simultaneously at shared sampling points. The resulting differences were below ±0.05 mg l^−1^, and statistical testing confirmed that there was no significant discrepancy between the two methods. Moreover, the internal consistency of the entire dataset was verified through Cronbach’s alpha (α = 0.793), and strong inter-point correlations and principal component (PC) structure confirmed that combining these datasets did not introduce inconsistency or bias in the results. Therefore, data from both techniques were considered reliable and directly comparable in subsequent analyses.

**Figure 2.**
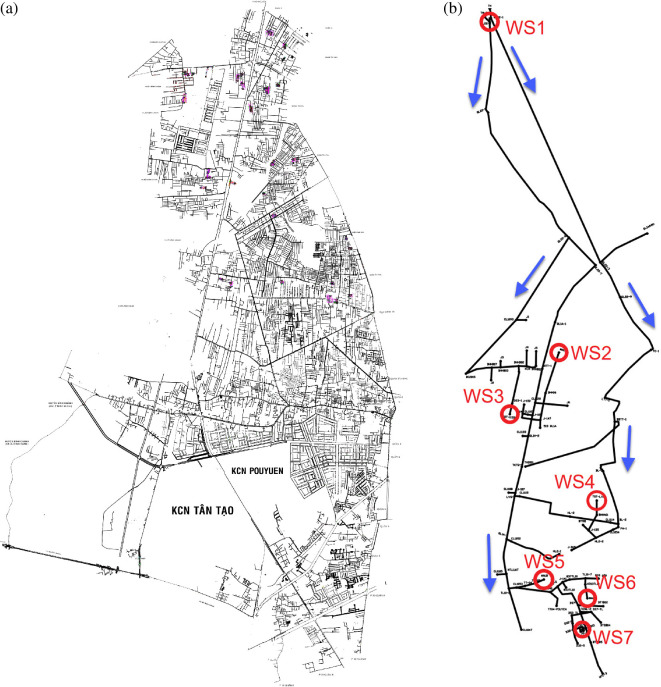
Water distribution network and RC sampling locations.

Figure 2 presents (i) the structural schematic of the Binh Tan District water distribution network and (ii) the simplified spatial layout of the seven RC sampling points (WS1–WS7) used in this study. The red circles labelled WS1–WS7 represent the actual locations of RC sampling points used in the study. This network is crucial for ensuring adequate water quality and safety throughout distribution systems.

Chlorine levels at WS1 through WS7 were monitored from June 2022 to December 2022. RC was measured at least once per day at all sampling points. At locations equipped with automatic monitoring stations (specifically WS3, WS4 and WS7), measurements were recorded multiple times per day, often at hourly intervals. However, to ensure consistency and comparability across all sites, we used only the daily value per day.

Furthermore, as data were collected between June and December, a period covering both dry and early wet seasons in southern Vietnam, seasonal factors such as temperature, rainfall and water demand may have influenced chlorine decay. Although our monthly aggregation smooths short-term variation, these broader seasonal dynamics could partially explain differences across stations and time. Future extensions could integrate seasonal or climatic variables into the models to improve predictive resolution.

The monthly averages presented in table 1 were derived from these standardized daily values. Additionally, table 1 includes both the monthly averages and pipeline lengths from each sampling point to WS1. This data collection process was carried out with the valuable support of the technical staff from Cho Lon Water Supply Joint Stock Company.

**Table 1.** Monthly RC levels at various measuring points (June 2022–December 2022).

date	WS1 (Tan Hiep water treatment plant)	WS2	WS3	WS4	WS5	WS6	WS7
Jun 22	1.05	0.63	0.55	0.52	0.76	0.48	0.35
Jul 22	1.00	0.31	0.33	0.31	0.38	0.25	0.23
Aug 22	0.90	0.62	0.61	0.56	0.46	0.55	0.53
Sep 22	1.00	0.58	0.41	0.38	0.30	0.31	0.28
Oct 22	1.10	0.36	0.59	0.51	0.91	0.56	0.48
Nov 22	0.90	0.56	0.53	0.54	0.41	0.45	0.42
Dec 22	0.90	0.39	0.49	0.58	0.63	0.46	0.34
distance (km)	—	14.94	16.14	18.35	18.92	20.89	21.07

The distance values in table 1 represent the cumulative length of the distribution pipeline from WS1 (the treatment plant) to each sampling point, measured along the actual pipeline path, not in straight-line distance. All sampling points are located at network monitoring junctions or intermediate chambers along the distribution grid, not directly at consumer premises. These points were chosen in consultation with the local utility (Cho Lon Water Supply JSC) based on hydraulic relevance and accessibility.

### 2.4. Data analysis

Statistical procedures for water quality and optimal efficiency water distribution systems are commonly discussed in the literature [26–30]. In the mathematical approach of the study, each statistical method is discussed not only to provide exploratory insights but also to determine the decision-making process in water quality monitoring and proactive chlorination strategies. In this sense, a few reminders may be appropriate before discussing the mathematical background of the study. Statistical analyses are performed using SPSS (Statistical Package for the Social Sciences) software. The following analyses are conducted.

*Cronbach’s alpha* is a data security analysis. It provides information about the network monitoring credibility and efficiency. In this sense, it ensures the internal consistency between sampling points by identifying stations (data) with inconsistent behaviour, unreliable or unnecessary. Cronbach’s alpha is a crucial reliability test used to assess the internal consistency of scales and equations with normally distributed variables [31]. This reliability measure is vital for researchers to ensure the dependability of their data. The alpha coefficient provides a quantitative assessment of reliability [32], categorizing it as moderate when the values fall between 0.41 and 0.60, good between 0.61 and 0.80 and highly reliable when the values range from 0.81 to 1.00 [33]. As one of the most frequently utilized methods for estimating internal consistency, Cronbach’s alpha coefficient stands out as the most widely used procedure for evaluating reliability in applied research, making it an essential tool for researchers aiming to validate their findings.

*Principal component analysis (PCA*) is a method of reducing a dataset belonging to a multidimensional space to smaller dimensions while preserving its basic properties. It is used to reduce the dimensionality of the data and to detect latent variables (components) influencing chlorine decay across the system. PCA helps to understand better underlying patterns, such as systemic factors or environmental influences, which impact chlorine dynamics in WDN. The process begins by selecting the appropriate line with the least average distance to all the points for a set of points in space. The most suitable line was then selected from among the orthogonal lines. This iteration was repeated until the variance of a new dimension fell below a certain threshold. At the end of the process, the bases of the linear space were created using the obtained lines. These base vectors are called the PCs. The PCs of the dataset were independent of each other.

*Binomial and Poisson distributions* play a crucial role in modelling RC levels in water distribution networks using statistical methods. The Binomial distribution is used to evaluate whether the measured RC at specific time intervals or spatial locations falls within acceptable limits, treating each measurement as a binary outcome (success/failure) with a constant probability of success and assuming independence between discrete observations [34]. This approach is particularly effective for assessing compliance in a fixed number of samples. The binomial formula is given by Sinharay [35]:


(2.1) $$P\left(k\right)=\left(\begin{array}{c} n\\ k \end{array}\right)p^{k}{\left(1-p\right)}^{n-k}$$

Here, *k* is the desired number of values from 0 to *n*, where *n* is the total number of measurements and *p* is the probability of the desired conditions.

On the other hand, the Poisson distribution is suitable for modelling the frequency of rare deviations in chlorine levels over a unit of discrete time or pipe length [36]. It is ideal for continuous monitoring systems, capturing low-probability events over many trials. Both distributions are valuable for statistically analysing the spatiotemporal variability of RC concentrations, detecting anomalies and optimizing quality control processes. Poisson formulation is given by Sinharay [35]:


(2.2) $$P\left(k\right)=\frac{\lambda^{k}e^{-\lambda }}{k!},$$

where *k* is the desired number of values and λ is the average success number.

*Regression analysis* was used to model the relationship between the dependent and independent variables (s). The model of this relationship not only reveals how the dependent variable changes (will change) according to the independent variable, but also indicates the strength of the change. Regression creates a model using a sample dataset and predicts future values based on this model. The consistency of the model belonging to the dataset obtained using an accurate and reliable method was specified using the correlation coefficient (*R*). The *R* rate was applied to detect the dual correlation of the data. Correlation coefficient:


(2.3) $$R=\frac{Cov(x,y)}{\sqrt{Var\left[x\right]Var[y]}}$$

where *x* (actual data) is the independent variable, *y* (predicted data) is the dependent variable, *cov(x,y*) is the covariance of *x; y*, *var*[*x*] and *var*[*y*] are the variances of *x* and *y*, respectively.

## 3. Results and discussion

Before presenting the modelling results, it is important to note that while chlorine concentration typically declines with increased distance from the source [1,37], the data revealed some notable deviations. For example, WS5 recorded a higher RC level than WS3, despite being located farther from WS1. This anomaly is attributed to local hydraulic dynamics: WS5 lies on a high-flow main line with faster turnover and lower water age, whereas WS3 is part of a peripheral sub-network where stagnation, low pressure and longer residence times contribute to accelerated chlorine decay [12,38]. These spatial inconsistencies underscore the importance of incorporating hydraulic parameters [39], such as flow velocity and pipe topology, into the interpretation of chlorine behaviour.

### 3.1. Reliability analysis

This study conducted a reliability analysis of the water samples along the water distribution system. Table 2 presents the item-total statistics for the water samples (WS). The terms ‘corrected item-total correlation’ and ‘Cronbach’s alpha if item deleted’ are standard outputs used in reliability analysis, specifically for computing internal consistency using Cronbach’s alpha.

**Table 2.** Item-total statistics for water sampling points.

	scale mean if item deleted	scale variance if item deleted	corrected item-total correlation	Cronbach’s alpha if item deleted
WS1	2.8429	0.362	0.065	0.824
WS2	3.3286	0.336	0.131	0.837
WS3	3.3200	0.267	0.946	0.706
WS4	3.3357	0.286	0.733	0.739
WS5	3.2714	0.214	0.536	0.808
WS6	3.3843	0.251	0.945	0.691
WS7	3.4457	0.279	0.749	0.733

Corrected item-total correlation refers to the correlation between each individual sampling point (WS1–WS7) and the sum of the remaining points. This reflects how well each item (each water sampling point’s RC) aligns with the overall structure of the dataset. A higher correlation indicates that the item contributes more consistently to the overall scale.

Cronbach’s alpha if item deleted shows what the Cronbach’s alpha value would be if that particular item were removed from the dataset. This helps identify whether any individual sampling point negatively affects the internal consistency of the overall scale.

Although WS1–WS7 are measurement points, they are treated here as parallel variables to assess the internal coherence of the chlorine removal measurements across stations. Therefore, these metrics help evaluate the reliability and agreement of the readings among all stations.

It can be seen from table 2 that the reliability of the models obtained increases from α = 0.793 when WS1 and WS2 stations are removed. We cannot infer from this table that WS1 and WS2 stations are related.

For the reliability analysis (Cronbach’s alpha), the RC values measured at each station per time point were summed to create a total scale score, consistent with standard scale-based reliability procedures. Consequently, the scale means reflect these summed totals rather than raw individual concentrations.

Cronbach’s alpha for all seven variables was 0.793, indicating good internal consistency. Removing WS1 or WS2 would slightly improve the overall reliability, whereas removing any other variable would decrease it.

From a practical perspective, these reliability patterns help utilities determine which sampling points consistently reflect system-wide chlorine behaviour. The relatively low consistency of WS1 and WS2 suggests these stations behave independently of the broader network. This can guide resource prioritization, where highly consistent points (WS3, WS6 and WS7) are prioritized for automated continuous monitoring, while less consistent ones may require targeted investigation for anomalies or localized issues.

### 3.2. Correlation analysis

Correlation analysis is applied to identify the strength and direction of relationships between sampling points. It allows researchers to determine binary strength points by monitoring. This step is essential in understanding whether certain monitoring stations provide redundant information or reflect shared behaviours, important for optimizing sensor placement and reducing operational costs in the water distribution system. Table 3 presents the correlation matrix for the water sampling points. The correlation analysis revealed strong relationships between several variables, particularly WS3, WS4, WS6 and WS7, which showed high positive correlations (*r *> 0.9). WS1 exhibited a strong negative correlation with WS4, WS6 and WS7.

**Table 3.** Correlation matrix for water sampling points.

	WS1	WS2	WS3	WS4	WS5	WS6	WS7
WS1	1	–0.554	–0.697	–0.850^a^	–0.397	–0.857^a^	–0.903^a^
WS2		1	0.631	0.596	0.110	0.570	0.587
WS3			1	0.926^a^	0.720^b^	0.955^a^	0.916^a^
WS4				1	0.657	0.962^a^	0.923^a^
WS5					1	0.731^b^	0.609
WS6						1	0.979^a^
WS7							1

^a^ Correlation is significant at the 0.01 level (two-tailed).

^b^ Correlation is significant at the 0.05 level (two-tailed).

High correlations among WS3, WS4, WS6 and WS7 indicate redundancy in sensor output, allowing utilities to consolidate monitoring efforts and reduce cost by installing fewer sensors without losing data fidelity. Conversely, the negative correlation between WS1 and other points suggests chlorine decay dynamics and hydraulic separation, requiring distinct monitoring protocols at the treatment outlet compared with distal zones.

The negative sign in Pearson’s correlation coefficient does not imply a problem with the system but rather reflects a statistical trend. It simply shows that when values at WS1 rise, values at some other locations may tend to decrease. In addition to this circumstance, Pearson’s correlation is limited to linear trends. Pearson’s correlation coefficient, also known as Pearson’s *R*, only captures linear relationships. If the actual relationship is nonlinear, or if the variables are influenced by external environmental factors (e.g. pipe distance, local consumption or pressure dynamics), the correlation may misrepresent the true underlying pattern.

In the different sampling contexts, WS1 represents treated water at the treatment plant outlet, while other stations represent different points in the WDN. Varying delays, decay rates or environmental effects along the distribution network could easily result in differing trends and even inverse correlations, particularly when monthly averages are used. However, our approach accounts for this variability through spatial clustering (PCA and hierarchical analysis) and regression models that incorporate both distance and site-specific behaviour. While monthly data were used for consistency, the framework is scalable to finer temporal resolutions, enabling predictive insights under both spatial heterogeneity and temporal fluctuations.

### 3.3. Factor analysis

Before conducting factor analysis, it is important to assess whether the dataset is suitable for such analysis. Two key indicators are used: Kaiser–Meyer–Olkin (KMO) measure of sampling adequacy and Bartlett’s test of sphericity measures.

KMO measure of sampling adequacy index indicates the proportion of variance among variables that might have been common variance. One can realize from that it is caused by underlying factors. It values range from 0 to 1. A KMO value above 0.6 is considered acceptable. In other words, if the KMO value is more than 0.6, it means that factor analysis can be performed.

Bartlett’s test of sphericity reviews whether the correlation matrix is significantly different from an identity matrix (i.e. one with no correlations among variables). A significant result (*p* < 0.05) indicates that the variables are correlated enough to justify factor analysis.

Factor analysis was performed to identify the underlying constructs at the water sampling sites. The KMO measure of sampling adequacy was 0.629, and Bartlett’s test of sphericity was significant (*p* < 0.001), indicating that the data were suitable for factor analysis.

Table 4 presents the results of the PCA, showing that three components with eigenvalues greater than 1 were extracted, cumulatively explaining 92.558% of the total variance in the data. The first component accounted for most of the variance (51.918%), while the second and third components contributed 23.761 and 16.878%, respectively.

**Table 4.** Total variance explained by principal components.

component	initial eigenvalues			extraction sums of squared loadings			rotation sums of squared loadings		
	total	% of variance	cumulative %	total	% of variance	cumulative %	total	% of variance	cumulative %
1	4.153	51.918	51.918	4.153	51.918	51.918	4.062	50.774	50.774
2	1.901	23.761	75.680	1.901	23.761	75.680	1.919	23.983	74.757
3	1.350	16.878	**92.558**	1.350	16.878	92.558	1.424	17.802	92.558

**Figure 3.**
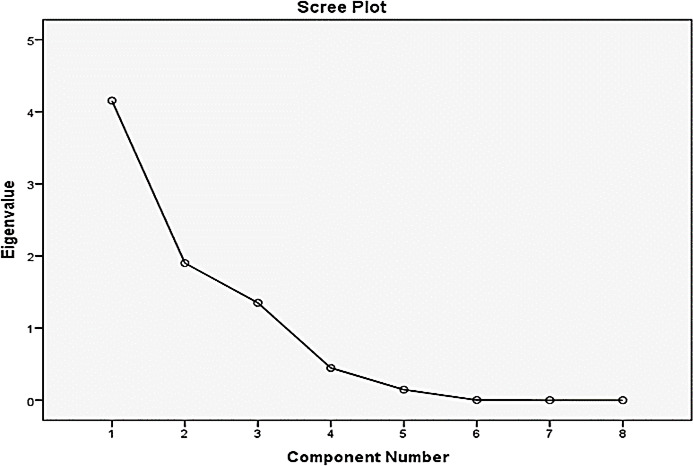
Scree plot of eigenvalues.

Figure 3 shows the scree plot of eigenvalues, which shows the number of variables (as an eigenvalue) that can form a cluster. The eigenvalues marked one and above are the eigenvalues that can explain the model. The three eigenvalues can explain the distance and chlorine discussion of the problem by over 92%. The eigenvalues were displayed to the reader using the rotation matrix displayed in table 4.

The rotated component matrix (table 5) shows that component 1 was primarily associated with WS3, WS4, WS6 and WS7. Component 2 is strongly related to WS1 and moderately related to WS5, whereas component 3 is mainly associated with WS2 and negatively related to distance. Here, the mentioned distance variable is normalized by the min–max normalization standard because of its effectiveness in harmony with the sources. Normalization is performed using the (*x − x*_min_)/(*x*_max_* − x*_min_) formula.

**Table 5.** Rotated component matrix. Bold values indicate the largest loading for each variable across components and are considered significant for interpretation.

variable	component		
	1	2	3
WS1		0.**925**	
WS2		–0.306	0.**877**
WS3	0.**969**		
WS4	0.**918**		
WS5	0.584	0.771	
WS6	0.**985**		
WS7	0.**928**		
distance		–0.560	–0.765

Table 5 points to clarify the distance-chlorine relationship problem in three axes. The axes are:

Axes1:0.969×WS3+0.918×WS4+0.584×WS5+0.985×WS6+0.928×WS7

**Figure 4.**
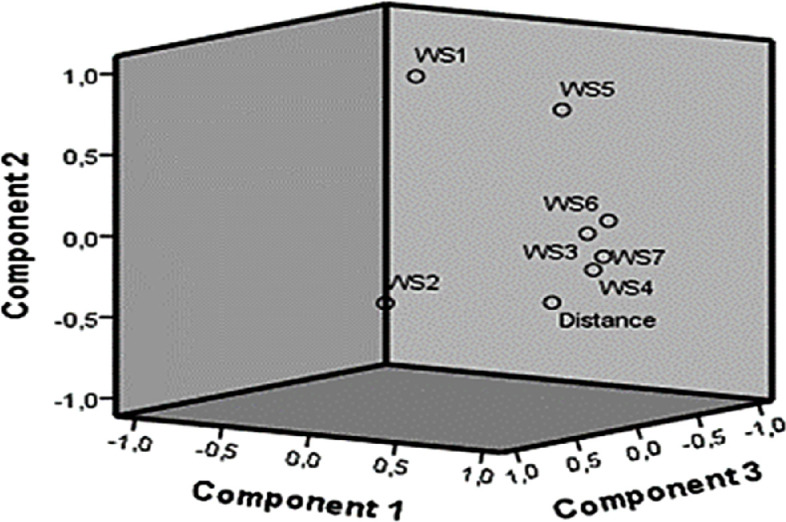
Component plot in rotated space

Figure 4 shows the component plot in the rotated space, visualizing the relationships between the variables and their associations with the extracted components. The plot confirms the strong association of WS3, WS4, WS6 and WS7 with component 1, whereas WS1 and WS5 were more closely related to component 2. WS2 and distance showed a strong relationship with component 3, but in opposite directions. This visualization helps interpret the factor structure and supports the findings from the rotated component matrix.

Factor groupings can be translated into operational sub-zones within the WDN. For example, the grouping of WS3, WS4, WS6 and WS7 under component 1 highlights a hydraulically coherent zone that may benefit from zonal chlorination control or shared booster management strategies. WS1 and WS5’s joint loading suggests a primary distribution corridor, potentially warranting separate pressure or flow regulation policies. Utilities can leverage this structure to optimize zonal re-chlorination or flushing intervals.

### 3.4. Hierarchical cluster analysis

Hierarchical clustering is used to optimize sensor placement and reduce work loss. It provides a spatial perspective on the similarity of sampling points. To further explore the relationships between variables, hierarchical cluster analysis was performed using the Ward method with squared Euclidean distances.

**Figure 5.**
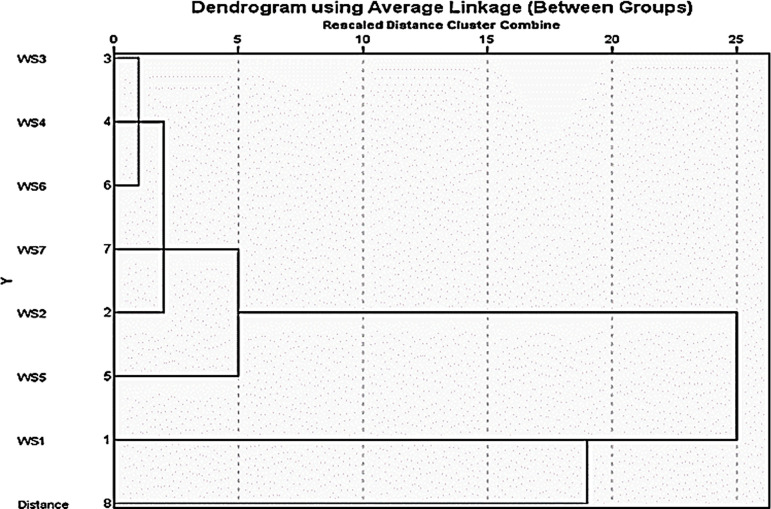
Dendrogram of water sampling points.

The dendrogram in figure 5 illustrates the hierarchical clustering of water sampling points. This shows that WS3, WS4, WS6 and WS7 formed a tight cluster, which aligns with their high correlations and loadings on the first PC. WS5 joins this cluster at a higher level, whereas WS1 and WS2 form separate clusters. This clustering structure supports the findings of the correlation and factor analyses, highlighting the distinct behaviour of WS1 and WS2 compared with the other variables.

These clusters provide a practical framework for regionalized maintenance, allowing network operators to target groups of similar stations for batch interventions, e.g. synchronized flushing or zone-specific dosing. The isolated cluster of WS1–WS2 implies the need for individualized operational thresholds, whereas the close clustering of WS3–WS7 allows for batch quality control strategies. It also helps streamline resource allocation and workforce scheduling.

### 3.5. Binomial and Poisson distributions

Table 6 presents the probability results of the binomial and Poisson distributions. In the Poisson distribution, the expected value is $\lambda =\frac{1}{n}\sum_{i=1}^{n}\lambda_{i}$. In table 6, λ=(3+3+2+4+5+6)/6.

**Table 6.** Binomial and Poisson distribution results.

station	observed (k)	binomial (P(k))		Poisson (P (kλ; and λ = 3.83)
		probability (%)	expected value	probability (%)
WS2	3	29.38	2.571	20.33
WS3	3	29.38	2.571	20.33
WS4	2	31.87	1.714	15.92
WS5	4	29.38	3.429	19.46
WS6	5	31.87	4.289	14.91
WS7	6	39.66	5.143	9.52

From a management perspective, binomial distribution results indicate the monthly compliance frequency of each station with the WHO-recommended RC range (0.2−0.5 mg l^−1^) over 7 months. This provides a basis for prioritizing quality assurance, intervention planning and regulatory reporting. The Poisson distribution, calculated using the average number of compliant months across all stations (*λ* = 3.83), enables the assessment of how statistically expected or unusual each station’s observed performance is. For instance, station WS7 maintained compliance in 6 out of 7 months (*k* = 6), which corresponds to a Poisson probability of 9.52%. This indicates that WS7 exhibited substantially above-average performance. Joint interpretation of binomial and Poisson outcomes guides risk-based monitoring adjustments, helping to ensure that high-performing yet statistically uncommon results are not mistakenly assumed to be inherently stable.

### 3.6. Regression analysis

Several regression models have been developed to understand the relationship between the water sampling points and distance.

WS4 and WS7 are critical stations within the network, strategically selected for their significance in monitoring RC levels. WS4 is centrally located, while WS7 is at the farthest end of the system. These points show a strong statistical correlation, reinforcing their suitability as output (dependent) variables in predictive models, while others serve as explanatory inputs (independent variables). Their selection enables early detection of chlorine fluctuations, allowing timely interventions to maintain water quality. This approach ensures effective, cost-efficient monitoring and supports data-driven decision-making within the system.

Table 7 presents the combined results of the parameter estimation and ANOVA for the statistical template in model 1. The parameter estimation section shows the coefficients for distance (a = 0.022), WS5 (b = −0.179) and WS6 (c = 1.049), along with their standard errors and 95% confidence intervals (95% CI). WS6 was the most significant predictor, and its confidence interval did not include zero. The ANOVA section indicates a regression sum of squares of 1.052 with three degrees of freedom (d.f.) and a residual sum of squares of 0.005 with 4 d.f., suggesting that the model accounts for a large proportion of the total variance (1.057).

**Table 7.** Statistical analysis of WS7 predictors. (a) Parameter estimation and (b) ANOVA.

(a) parameter estimation					(b) ANOVA results			
parameter	estimate	s.e.	95% CI		source	sum of squares	d.f.	mean squares
			lower bound	upper bound				
a (distance)	0.022	0.038	–0.085	0.128	regression	1.052	3	0.351
b (WS5)	–0.179	0.095	–0.444	0.085	residual	0.005	4	0.001
c (WS6)	1.049	0.156	0.617	1.481	uncorrected total	1.057	7	

Model 1 demonstrates high explanatory power of 96.2% (√*R*² = 0.926), indicating that the variance in WS7 is explained by the predictors. The model also showed good reliability, with a Cronbach’s alpha (α) of 0.763. These metrics suggest that the combination of distance, WS5 and WS6 effectively predicts WS7, with WS6 likely being the strongest contributor.


Model1:WS7=f(Distance, WS5, WS6)=a×Distance+b×WS5+c×WS6

According to table 8, the difference between the sum of squares of the variance between the variables and their residuals highlights the significance of the model for model 2.

**Table 8.** Statistical analysis of WS4 predictors. (a) Parameter estimation and (b) ANOVA.

(a) parameter estimation					(b) ANOVA results			
parameter	estimate	s.e.	95% CI		source	sum of squares	d.f.	mean squares
			lower bound	upper bound				
a (distance)	0.046	0.066	–0.136	0.228	regression	1.697	3	0.566
b (WS2)	0.028	0.201	–0.529	0.585	residual	0.015	4	0.004
c (WS3)	0.872	0.236	0.218	1.526	uncorrected total	1.713	7	

Model 2 offers a meaningful approach to solving the problem, with the relationship between distance, WS2 and WS3 showing a strong correlation of 86.7% (√*R*² = 0.751) with the actual data. Additionally, the model demonstrated good reliability, as evidenced by Cronbach’s alpha of 0.749. This indicates that the model effectively captured the variance in the data.


Model 2:WS4=f(Distance, WS2, WS3)=a×Distance+b×WS2+c×WS3

As shown in table 9, the difference between the sum of squares of the variance between variables and their residuals underscores the significance of the model for model 3. These findings suggest that WS3, WS6 and WS7 were effective predictors of WS4.

**Table 9.** Statistical analysis of WS4 predictors. (a) Parameter estimation and (b) ANOVA.

(a) parameter estimation					(b) ANOVA results			
parameter	estimate	s.e.	95% CI		source	sum of squares	d.f.	mean squares
			lower bound	upper bound				
a (WS3)	1.007	0.636	–0.758	2.772	regression	1.699	3	0.566
b (WS6)	0.415	0.751	−1.670	2.499	residual	0.014	4	0.003
c (WS7)	–0.538	0.562	−2.097	1.022	uncorrected total	1.713	7	

Model 3 offers a robust approach to solving the problem, with the relationship between WS3, WS6 and WS7 showing a strong correlation of 88.0% (√*R*² = 0.775) with the actual data. Moreover, the model showed outstanding reliability, as indicated by Cronbach’s alpha of 0.967. This indicates that the model effectively captured the variance in the data.


Model 3:WS4=f(WS3, WS6, WS7)=a×WS3+b×WS6+c×WS7

Table 10 highlights the significance of the model by comparing the sum of the squares of the variance between the variables and their residuals for Model 4. The parameter estimation shows the coefficients for distance (0.047) and WS6 (0.784), with WS6 being the most significant predictor, as its confidence interval does not include zero. The ANOVA results revealed a regression sum of squares of 1.047 with 2 d.f. and a residual sum of squares of 0.010 with 5 d.f., suggesting that the model explains a substantial portion of the total variance (1.057).

**Table 10.** Statistical analysis of WS7 predictors—parameter estimation (a) and ANOVA (b).

(a) parameter estimation					(b) ANOVA results			
parameter	estimate	s.e.	95% CI		source	sum of squares	d.f.	mean squares
			lower bound	upper bound				
a (distance)	0.047	0.044	–0.067	0.160	regression	1.047	2	0.524
B (WS6)	0.784	0.080	0.578	0.990	residual	0.010	5	0.002
					uncorrected total	1.057	7	

Model 4 provides a robust solution, with the relationship between distance and WS6 showing a strong correlation of 92.7% (√*R*² = 0.86) with the actual data and a Cronbach’s alpha of 0.952. These findings suggest that distance and WS6 are effective predictors of WS7, with WS6 being the most influential.


Model 4:WS7=f(Distance, WS6)=a×Distance+b×WS6

Table 11 illustrates the combined results of parameter estimation and ANOVA for model 5. The parameter estimation section shows the coefficients for distance (0.044) and WS3 (0.902), with their respective standard errors and 95% CIs. WS3 is the most significant predictor because its confidence interval does not include zero. The ANOVA results indicated a regression sum of squares of 1.697 with 2 d.f. and a residual sum of squares of 0.015 with 5 d.f., suggesting that the model explains a substantial portion of the total variance (1.713).

**Table 11.** Statistical analysis of WS4 predictors. (a) Parameter estimation and (b) ANOVA.

(a) parameter estimation					(b) ANOVA results			
parameter	estimate	s.e.	95% CI		source	sum of squares	d.f.	mean squares
			lower bound	upper bound				
a (distance)	0.044	0.058	–0.104	0.193	regression	1.697	2	0.849
b (WS3)	0.902	0.092	0.664	1.139	residual	0.015	5	0.003
					uncorrected total	1.713	7	

Model 5 demonstrates strong explanatory power with √*R*² = 0.750, indicating that 86.6% of the variance in WS4 is accounted for by the predictors. Additionally, the model showed high reliability, as evidenced by Cronbach’s alpha of 0.922. These metrics suggest that Distance and WS3 are effective predictors of WS4, with WS3 being the most influential.


Model 5:WS4=f(Distance, WS3)=a×Distance+b×WS3

The results of this study offer actionable insights for enhancing water distribution network (WDN) management. For example, model 1 can be embedded in real-time SCADA systems to predict RC at WS7, allowing proactive interventions (e.g. chlorine dosing or pipe flushing) before levels fall below acceptable thresholds. Models relying on spatial parameters like ‘distance’ provide a low-cost, data-light alternative when sensor data are temporarily unavailable. Similarly, the hierarchical clustering results, which group WS3, WS4, WS6 and WS7 into a single high-correlation cluster, provide a clear basis for designing unified monitoring or intervention protocols within this zone, thereby reducing operational complexity. The PCA further reveals that WS2 and WS1 exhibit distinct chlorine behaviour, suggesting their use as sentinel points to detect localized anomalies or disruptions in the network. Moreover, the role of distance as a consistent predictor in multiple models supports its utility in infrastructure planning, specifically in deciding optimal locations for booster chlorination stations or scheduling pipe replacements where chlorine decay is most rapid. These examples underscore how the statistical models developed in this study can directly support system optimization, proactive maintenance and regulatory compliance in urban water supply systems.

Real-time RC prediction plays a crucial role in ensuring continuous monitoring of water quality, even when certain sensors are offline or undergoing maintenance. By leveraging predictive models, utilities can maintain operational awareness and avoid gaps in data collection. Additionally, determining intervention points allows for the early detection of critical chlorine drops, enabling timely re-chlorination or flushing to preserve water safety. A significant advantage of these models is their cost-effective monitoring capability, as they reduce the necessity of deploying sensors at every location while still providing reliable estimates. This optimization not only lowers costs but also enhances efficiency in resource allocation. They can also be used in periodic assessments to evaluate water quality stability, helping authorities make informed decisions regarding maintenance and intervention strategies.

While this study focuses on Binh Tan District, the statistical modelling framework is not inherently site-dependent and can be adapted to other urban water distribution systems with similar data availability. The combination of PCA, regression and cluster analysis constitutes a universal methodology that can be used wherever RC data and spatial pipeline information are available. For instance, urban utilities in different countries can replicate this approach by monitoring chlorine levels across representative sampling nodes, applying PCA to identify dominant patterns in water quality and using regression to predict RC fluctuations in under-monitored zones. Hierarchical clustering can similarly guide regional monitoring strategies or risk-based resource allocation. Importantly, these methods do not rely on fixed chlorine concentrations or treatment practices unique to Vietnam, making them highly transferable. Future studies can test the portability of these models by validating them in other cities with different network complexities and environmental conditions. Such cross-site validation would further strengthen the case for adopting this framework as a scalable decision-support tool in global water safety management.

## 4. Conclusion

This study successfully characterized the chlorine dynamics of the water distribution system in the Binh Tan District through comprehensive statistical analysis. Key findings included strong internal consistency, with an overall Cronbach’s alpha of 0.793, indicating good reliability across sampling points. The individual regression models also showed high reliability, with α values ranging from 0.749 to 0.967.

Distinct spatial patterns were identified, with three PCs explaining 92.558% of the total variance. Hierarchical analysis revealed clear clustering of sampling points (WS3, WS4, WS6 and WS7), while WS1 and WS2 exhibited distinct behaviour from the other sampling points.

The study also achieved success in predictive modelling, developing five regression models with high explanatory power (*R*² = 0.750–0.962). Model 1 demonstrated the highest predictive power for WS7, with a √*R*² of 0.926. Distance and specific sampling points have been proven to be effective predictors.

From a management perspective, these results support strategic monitoring point selection and enable the prediction of chlorine levels at different points. These findings can help optimize chlorination practices and monitoring strategies, providing a robust framework for water quality management and system optimization in similar urban distribution networks.

Despite the strengths of this study, certain limitations should be acknowledged. Factors such as water consumption patterns, pH variations, temperature fluctuations and operational scenarios may influence chlorine dynamics but were not explicitly incorporated into the analysis. Future research could integrate these variables to further refine predictive models and enhance the accuracy of chlorine monitoring strategies, ensuring a more comprehensive approach to water quality management.

## Data Availability

The datasets used in this study have been provided as supplementary material and are available in the Dryad repository [40]. Additionally, the data can be obtained upon reasonable request from the Cho Lon Water Supply Joint Stock Company, subject to their data-sharing policy. The authors will facilitate access to the data while ensuring the confidentiality of sensitive information.
